# Supplementary material for: An optogenetic method for the controlled release of single molecules
Source: Nat Methods. 2024 Mar 8;21(4):666–72. doi: 10.1038/s41592-024-02204-x (PMC11009104; doi:10.1038/s41592-024-02204-x)
Supplement: Supplementary file 1 — Supplementary Figs. 1–5 and primer list table. [file 41592_2024_2204_MOESM1_ESM.pdf]

nature methods

Article https://doi.org/10.1038/s41592-024-02204-x

An optogenetic method for the controlled release of single molecules

In the format provided by the authors and unedited

Supplementary Figures:

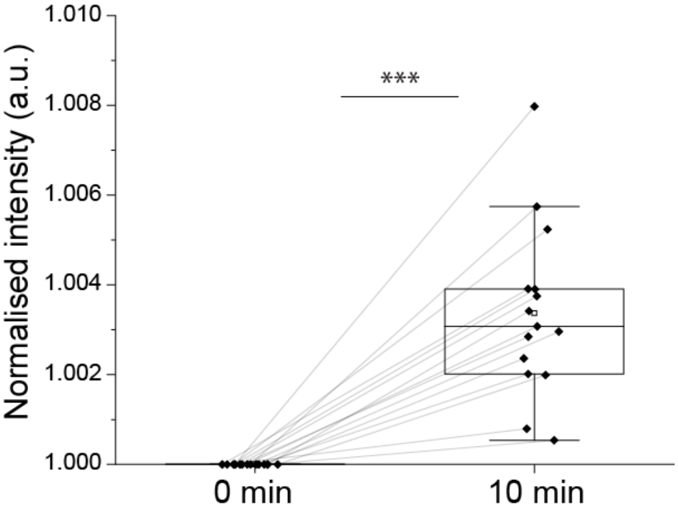

Supplementary Figure 1. Release of FGF2-GFP in the cytosol. Quantification of cytosolic FGF2-GFP before (1 ± 0) and 10 min after UV Illumination (1.05 ± 0.02) of CHO cells expressing TMEM-PhoCl-FGF2-GFP, N = 1, n = 14. Significance was tested using two-tailed paired sample sign test ($p = 10^{-4}$). Data in the legend are presented as mean ± SD. N = number of biological replicates, n = number of cells, SD = standard deviation. The center line in the box plot represents the median, the box represents the 25%-75% of the data, whiskers represent 1.5 interquartile range, the square box among the data points represents the mean.

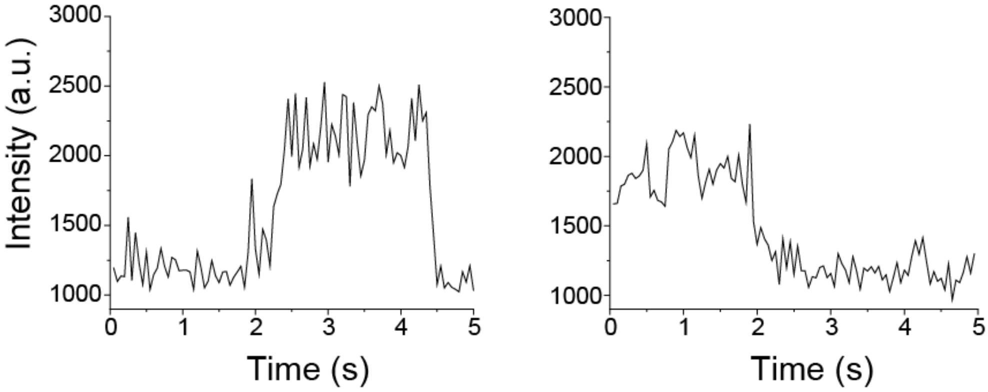

Supplementary Figure 2. Single FGF2-GFP at the plasma membrane. Time traces of fluorescence emission from FGF2-GFP molecules on the plasma membrane**.**

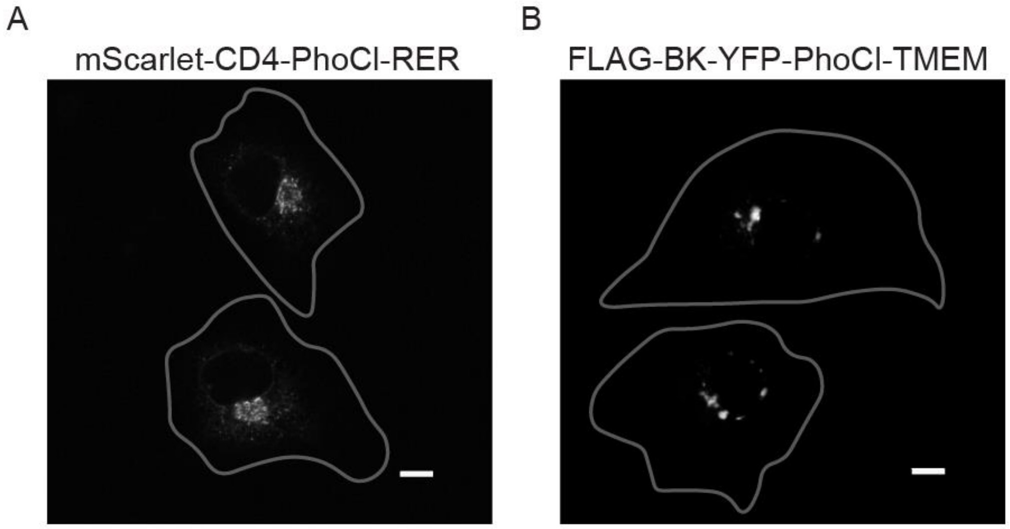

Supplementary Figure 3. Golgi localization of PhoCl caged proteins. (a) CV1 cells expressing mScarlet-CD4-PhoCl-RER **(b)** CV1 cells expressing FLAG-BK-YFP-PhoCl-TMEM. N = 3. Scale bars are 10 μm.

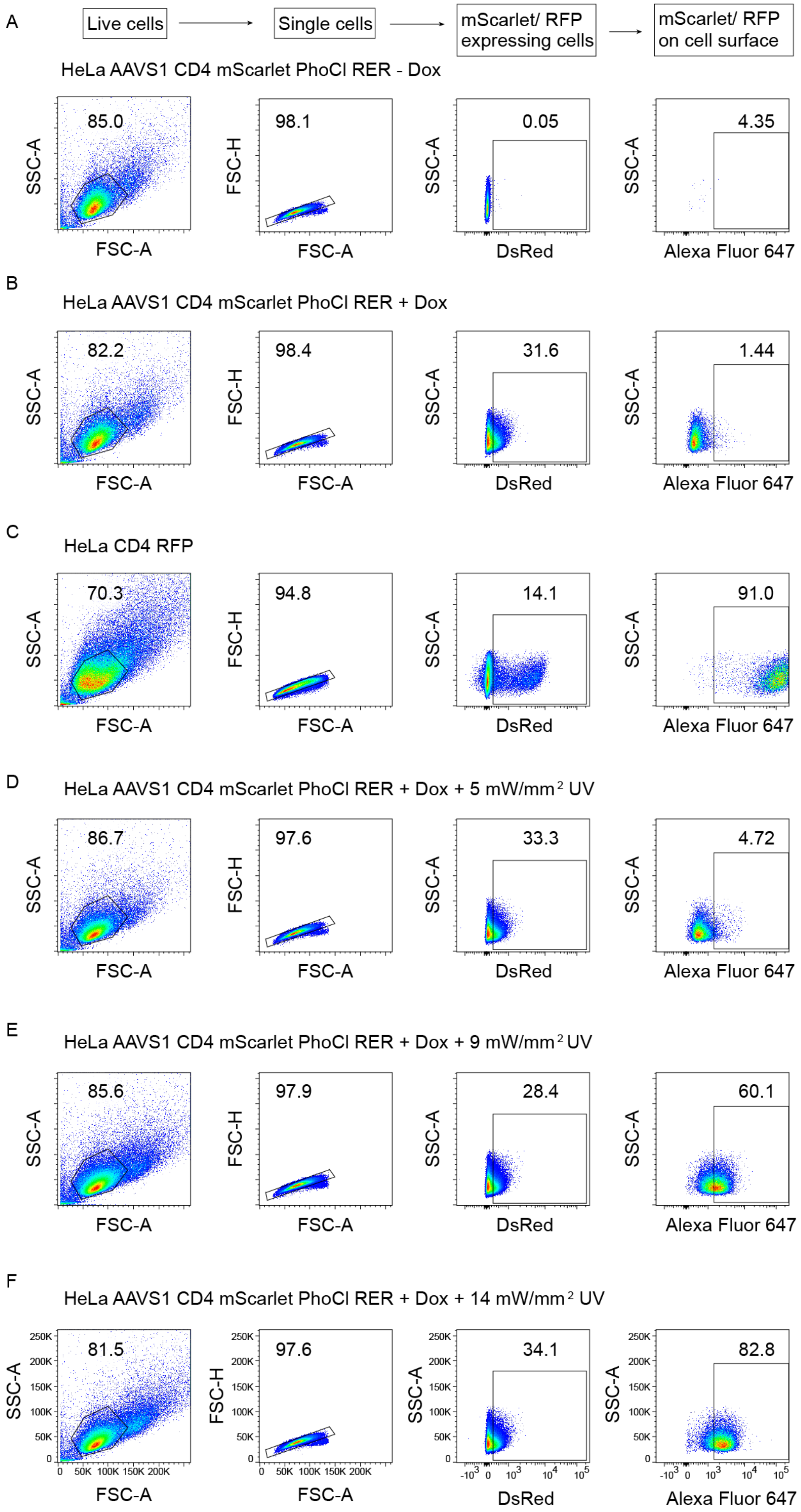
A
Live cells
Single cells
mScarlet/ RFP expressing cells
mScarlet/ RFP on cell surface
HeLa AAVS1 CD4 mScarlet PhoCl RER - Dox
85.0
98.1
0.05
4.35
B
HeLa AAVS1 CD4 mScarlet PhoCl RER + Dox
82.2
98.4
31.6
1.44
C
HeLa CD4 RFP
70.3
94.8
14.1
91.0
D
HeLa AAVS1 CD4 mScarlet PhoCl RER + Dox + 5 mW/mm² UV
86.7
97.6
33.3
4.72
E
HeLa AAVS1 CD4 mScarlet PhoCl RER + Dox + 9 mW/mm² UV
85.6
97.9
28.4
60.1
F
HeLa AAVS1 CD4 mScarlet PhoCl RER + Dox + 14 mW/mm² UV
81.5
97.6
34.1
82.8
SSC-A
FSC-H
FSC-A
DsRed
Alexa Fluor 647
250K
200K
150K
100K
50K
0
50K
100K
150K
200K
-10³
10³
10⁴
10⁵

Supplementary Figure 4. UV dose response of uncaging of mScarlet-CD4-PhoCl-RER. (a) Representative negative control, HeLa cells without doxycycline (Dox)-based expression of mScarlet-CD4-PhoCl-RER, and the gating strategy used for the flow cytometry experiments. Briefly, live cells were selected from which single cells were gated for. Next, cells expressing mScarlet-CD4-PhoCl-RER or CD4-mRFP (positive control) were selected for. In the last step, cells with membrane localized mScarlet-CD4 or CD4-mRFP detected by AF647 labeled anti-RFP antibody were selected. **(b)** Representative flow cytometry results from negative control, HeLa cells expressing mScarlet-CD4-PhoCl-RER under doxycycline but without UV treatment. **(c)** Representative flow cytometry results from positive control, HeLa cells overexpressing CD4-mRFP. **(d, e, f)** Representative flow cytometry results of HeLa cells expressing mScarlet-CD4-PhoCl-RER under doxycycline with increasing doses of UV treatment.

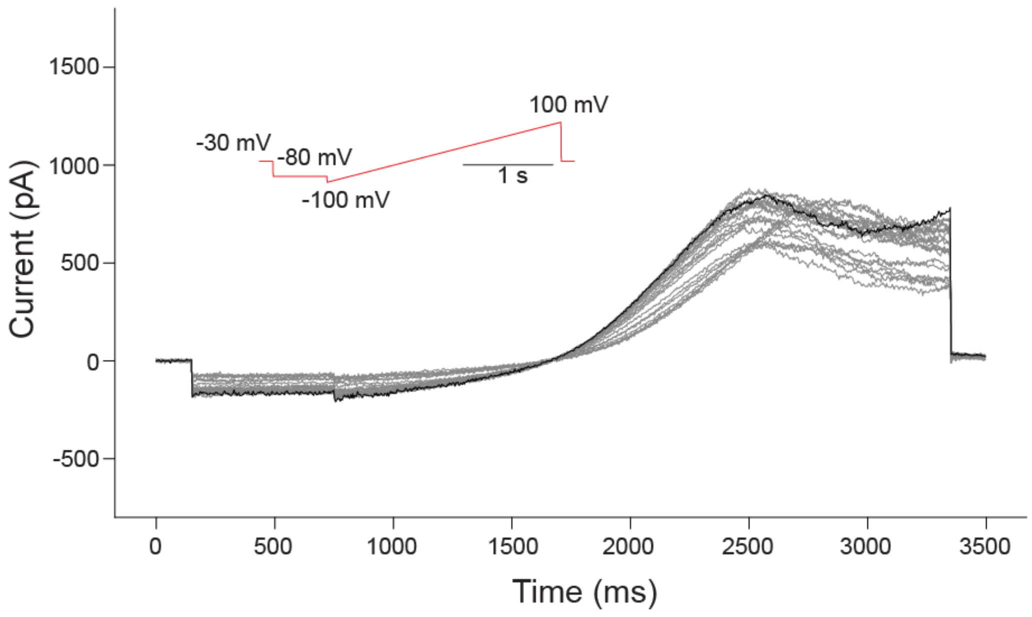

Supplementary Figure 5. Current kinetics of optogenetically released VRAC. Example ramp current traces from optogenetically released VRACs for 4 min upon switching bath solution to hypotonicity with maximal developed current trace shown in black. Ramp protocol shown red in inset. Note the fast inactivation at inside-positive voltages typical for LRRC8A/E-composed VRACs.

Primer list:

	Construct name	Fragment name		Primer sequence (5'-3')
1	TMEM-PhoCl-FGF2-GFP	TMEM115	For	aaaaaaa gctagc gccgccgcgatcg
			Rev	ttttttt tgtaca cagcgtcgggggagc
2	TMEM-PhoCl-FGF2-GFP	PhoCl	For	aaaaaaa ctcgag gtgatccctgactacttcaagcag
			Rev	ttttttt aagctt ggtacctccacctccccgt
3	TMEM-PhoCl-FGF2-GFP	FGF2-GFP	For	aaaaaaa gaattc aa accatggcagccggg
			Rev	ttttttt gtcgac ttacttgtacagctcgtccatgc
4	mScarlet-CD4-PhoCl-RER (Fig2B,C)	mScarlet - CD4-PhoCl2c-RER	For	tagtcagctgacgcgtgctagcggatccatgtggcctcttgtt
			Rev	ttatcgatgcggccgcgctagctctagattagctggcgaaggc
5	mScarlet-CD4-PhoCl-RER_stable cells (Fig2D,E)	Tet promotor	For	ctccaaggccttaattcgaaactagttaatgtgagttagctcactcattaggcac
			Rev	ggcgatctgacggttcactaaac
6	mScarlet-CD4-PhoCl-RER_stable cells (Fig2D,E)	mScarlet-CD4-PhoCl2c-RER	For	gtttagtgaaccgtcagatcgccgccaccatgtggcctcttgttgctgc
			Rev	tttgtaataaccgcggaatacgcgtttagctggcgaaggcttttcc
7	FLAG-BK-YFP-PhoCl-TMEM	FLAG-BK_fragment 1	For	atcctctagtcagctgacgcgtgaaccggtgccaccatggtgcatcaccatca ccat
			Rev	gacacgctgaacttgtggccgttta
8	FLAG-BK-YFP-PhoCl-TMEM	BK_fragment 2	For	taaacggccacaagttcagcgtgtc
			Rev	gtcgggtatgacgctgccgccgccgccaccggtaagccgctcttcctgcac
9	CACNA1E-eGFP-PhoCl-TMEM115	PhoCl2c_PCR 1	For	gcatggacgagctgtacaagaccggtggcggcggcggcagcgtcataccc gactacttcaagcagag
			Rev	tctagggtactttgtgaaca
10	CACNA1E-eGFP-PhoCl-TMEM115	PhoCl2c_PCR 2	For	gcatggacgagctgtacaag
			Rev	tgtgccgcctccgcctgtgccgcctccgcctctagggtactttgtgaaca
11	CACNA1E-eGFP-PhoCl-TMEM115	TMEM115	For	ggcggaggcggcacaggcggaggcggcacaatgcaacgtgccctgcca
			Rev	tcgacaagcttatcgatgcggccgcgctagcttacagcgtcgggggagctgc
12	LRRC8A-GFP-PhoCl-TMEM	LRRC8A_fragment1	For	atcctctagtcagctgacgcgtgaaccggtgccaccatgattccggtgacaga gctc
			Rev	ggatctccttgatgtcggtgaactt
13	LRRC8A-GFP-PhoCl-TMEM	LRRC8A_fragment2	For	aagttcaccgacatcaaggagatcc
			Rev	gtcgggtatgacgctgccgccgccgccaccggtcttgtacagctcgtccatgc c

14	LRRC8A-GFP-alfa-PhoCl-TMEM	Alfa_tag	For	aagttcaccgacatcaaggagatcc
			Rev	ggatctccttgatgtcggtgaactt
15	LRRC8A-PhoCl-TMEM	LRRC8A_fragment2	For	aagttcaccgacatcaaggagatcc
			Rev	atgacgctgccgccgccgccacccgcggcctgctccttgtcagc
16	TMEM-PhoCl-IRAK4-mScarlet	TMEM-PhoCl	For	tttttggaggcctaggctacgcgtgccaccatgcaacgt
			Rev	actaccaccagaacctccacttcctcctctagggtactttgtgaaca
17	TMEM-PhoCl-IRAK4-mScarlet	IRAK4-mScarlet	For	ggaggaagtggaggttctggtggtagtatgaacaagccgttgacaccat
			Rev	tgcaggtcgactctagagtcgcggccgctttacttgtacagct
