# Supplementary material for: An optogenetic method for the controlled release of single molecules
Source: Nat Methods. 2024 Mar 8;21(4):666–72. doi: 10.1038/s41592-024-02204-x (PMC11009104; doi:10.1038/s41592-024-02204-x)
Supplement: Supplementary file 2 — Reporting Summary [file 41592_2024_2204_MOESM2_ESM.pdf]

nature portfolio

Corresponding author(s): Helge Ewers

Last updated by author(s): Nov 27, 2023

# Reporting Summary

Nature Portfolio wishes to improve the reproducibility of the work that we publish. This form provides structure for consistency and transparency in reporting. For further information on Nature Portfolio policies, see our Editorial Policies and the Editorial Policy Checklist.

## Statistics

For all statistical analyses, confirm that the following items are present in the figure legend, table legend, main text, or Methods section.

| n/a | Confirmed | |
|---|---|---|
| ☐ | ☒ | The exact sample size (*n*) for each experimental group/condition, given as a discrete number and unit of measurement |
| ☐ | ☒ | A statement on whether measurements were taken from distinct samples or whether the same sample was measured repeatedly |
| ☐ | ☒ | The statistical test(s) used AND whether they are one- or two-sided<br>*Only common tests should be described solely by name; describe more complex techniques in the Methods section.* |
| ☒ | ☐ | A description of all covariates tested |
| ☒ | ☐ | A description of any assumptions or corrections, such as tests of normality and adjustment for multiple comparisons |
| ☐ | ☒ | A full description of the statistical parameters including central tendency (e.g. means) or other basic estimates (e.g. regression coefficient) AND variation (e.g. standard deviation) or associated estimates of uncertainty (e.g. confidence intervals) |
| ☐ | ☒ | For null hypothesis testing, the test statistic (e.g. *F*, *t*, *r*) with confidence intervals, effect sizes, degrees of freedom and *P* value noted<br>*Give P values as exact values whenever suitable.* |
| ☒ | ☐ | For Bayesian analysis, information on the choice of priors and Markov chain Monte Carlo settings |
| ☒ | ☐ | For hierarchical and complex designs, identification of the appropriate level for tests and full reporting of outcomes |
| ☒ | ☐ | Estimates of effect sizes (e.g. Cohen's *d*, Pearson's *r*), indicating how they were calculated |

*Our web collection on statistics for biologists contains articles on many of the points above.*

## Software and code

Policy information about availability of computer code

| | |
|---|---|
| Data collection | Metamorph 7.8.6.0, Micromanager 2.0, NIS-elements 5.11.01, Axograph Scientific 1.7, Patch Master v2x91 |
| Data analysis | Fiji 1.54f, Cell Profiler 4.2.1, IgorPro 9.0, OriginLab 2022b, R 4.2.2, FlowJo v10.10.0 |

For manuscripts utilizing custom algorithms or software that are central to the research but not yet described in published literature, software must be made available to editors and reviewers. We strongly encourage code deposition in a community repository (e.g. GitHub). See the Nature Portfolio guidelines for submitting code & software for further information.

## Data

Policy information about availability of data

All manuscripts must include a data availability statement. This statement should provide the following information, where applicable:

- Accession codes, unique identifiers, or web links for publicly available datasets
- A description of any restrictions on data availability
- For clinical datasets or third party data, please ensure that the statement adheres to our policy

Source data has been provided in excel files. Original images and current traces are too big to upload and are available upon request from the corresponding author.

## Research involving human participants, their data, or biological material

Policy information about studies with human participants or human data. See also policy information about sex, gender (identity/presentation), and sexual orientation and race, ethnicity and racism.

| | |
|---|---|
| Reporting on sex and gender | N/A |
| Reporting on race, ethnicity, or other socially relevant groupings | N/A |
| Population characteristics | N/A |
| Recruitment | N/A |
| Ethics oversight | N/A |

Note that full information on the approval of the study protocol must also be provided in the manuscript.

# Field-specific reporting

Please select the one below that is the best fit for your research. If you are not sure, read the appropriate sections before making your selection.

☒ Life sciences ☐ Behavioural & social sciences ☐ Ecological, evolutionary & environmental sciences

For a reference copy of the document with all sections, see nature.com/documents/nr-reporting-summary-flat.pdf

# Life sciences study design

All studies must disclose on these points even when the disclosure is negative.

| | |
|---|---|
| Sample size | The sample size varied depending on the type of experiments and is given in the figure legends for all experiments contained in this study. No pre study sample size calculations were performed. The sample size was determined based on the experimental design, the duration of the full experiment in a day and the time required for imaging live samples without stressing them. The electrophysiology experiments were limited by the number of successful patch clamps possible per day. The arbitrarily chosen sample size was sufficient to show significant difference. |
| Data exclusions | To restrict the analysis to fluorescent puncta that were bonafide Myddosomes, we perform the following normalization procedure. The intensities of MyD88, IRAK4 and pIKK at the 1% quantile were used as the minimum for normalization. We normalized the intensities using the equation: Norm. Int = (Intensity - quantile(0.01))/(quantile(0.99) - quantile(0.01)). We then rounded normalized intensities to 2 decimals. If puncta had negative intensities (intensity value < 0) after normalisation, they were removed from the analysis, to ensure the analysis of pIKK staining was restricted to segmented puncta that most likely were true Myddosome signaling complexes. To confirm this analysis did not possibly exclude true Myddosomes, we performed manual inspection of the segmented images and found this procedure only resulted 1 segmented punctum out of 114894 puncta being excluded from further data analysis visualization.<br>For data visualization purpose, the violin plots only show pIKK normalized intensity between 0-3, value > 3 were not shown on the plots but were included in calculating the mean. |
| Replication | Biological replicates varied depending on the type of experiments and is given in the figure legends for all experiments contained in this study. |
| Randomization | There are two categories in the study: control and UV treated samples. Cells were randomly assigned a category before application of UV, analysed and plotted for the final experiments through automated pipeline. Electrophysiology experiments, due to technical limitations (use of LED or lamps for UV illumination) were performed such that whole dishes were considered either illuminated or not illuminated (control samples). In this setup randomization of samples was not possible. |
| Blinding | All cells in predetermined control and UV treated groups were pooled together, analysed and plotted in an automated pipeline. |

# Reporting for specific materials, systems and methods

We require information from authors about some types of materials, experimental systems and methods used in many studies. Here, indicate whether each material, system or method listed is relevant to your study. If you are not sure if a list item applies to your research, read the appropriate section before selecting a response.

## Materials & experimental systems

| n/a | Involved in the study |
|---|---|
| ☐ | ☒ Antibodies |
| ☐ | ☒ Eukaryotic cell lines |
| ☒ | ☐ Palaeontology and archaeology |
| ☒ | ☐ Animals and other organisms |
| ☒ | ☐ Clinical data |
| ☒ | ☐ Dual use research of concern |
| ☒ | ☐ Plants |

## Methods

| n/a | Involved in the study |
|---|---|
| ☒ | ☐ ChIP-seq |
| ☐ | ☒ Flow cytometry |
| ☒ | ☐ MRI-based neuroimaging |

# Antibodies

Antibodies used

anti-RFP (supplier: Chromotek, clone nr.: 5F8, lot nr.: 11041)
anti-FLAG (supplier: Sigma-Aldrich, product nr.: F1804, batch nr.: SLCK5688)
anti-phospho-IKK (Supplier: Cell Signaling Technology, #2697)
Nano-secondary AF647 (Chromotek, #srbAF647-1-100)
FluoTag-X4 anti-GFP conjugated to Atto488 (NanoTag Biotechnologies, #N0304-At488-L)
FluoTag-X2 anti-mScarlet-i conjugated to Atto565 (NanoTag Biotechnologies, #N1302-At565-L)

Validation

anti-RFP (supplier: Chromotek, clone nr.: 5F8, lot nr.: 11041): Antibody was tested against CD4mRFP expressing live cells in TIRF microscopy in the lab. In the company website, immunofluorescence images of cells expressing MannosidaseII-tdTomato (Golgi) show good colocalisation with the antibody staining.

anti-FLAG (supplier: Sigma-Aldrich, product nr.: F1804, batch nr.: SLCK5688): Antibody was tested against Flag-BK expressing live cells in TIRF microscopy in the lab. The company website cites https://doi.org/10.1186/s12985-016-0610-7 for the use of the antibody for immunofluorescence where cells expressing glycoproteins gD-flag/gM-flag were costained with anti-FLAG.

anti-phospho-IKK (Supplier: Cell Signaling Technology, #2697): The antibody has been validated for WB, immunohistochemistry, and Flow cytometry by the company. For example, Flow cytometry example shows clear difference between control and, TPA and LPS treated THP cells using the antibody.

FluoTag-X4 anti-GFP conjugated to Atto488 (NanoTag Biotechnologies, #N0304-At488-L): Company website show multiple examples images of immunostaining. Examples include cells expressing Nup98-GFP (nuclear pore protein, colocalization with GFP) and TOM70-nfGFP-BFP(mitochondria and colocalization with BFP).

FluoTag-X2 anti-mScarlet-i conjugated to Atto565 (NanoTag Biotechnologies, #N1302-At565-L): Company website show multiple example images of immunostaining. Examples include cells expressing mScarlet-i-tubulin (microtubules, colocalization with mScarlet) and TOM70-nfmScarlet-BFP (mitochondria).

# Eukaryotic cell lines

Policy information about cell lines and Sex and Gender in Research

Cell line source(s)

CV1 from ATCC
HEK293T from Deutsche Sammlung von Mikroorganismen und Zellkulturen, Germany
CHO-K1 from ATCC
EL-4 from Clontech
HeLa from ATCC
HeLa-EM2 expressing rtTA2-M2 were a gift from Manfred Gossen laboratory. They are modified HeLa cells from ATCC.

Authentication

Cell lines were not authenticated

Mycoplasma contamination

Cells were tested negative for mycoplasma.

Commonly misidentified lines
(See ICLAC register)

Not used

## Flow Cytometry

### Plots

Confirm that:

☒ The axis labels state the marker and fluorochrome used (e.g. CD4-FITC).

☒ The axis scales are clearly visible. Include numbers along axes only for bottom left plot of group (a 'group' is an analysis of identical markers).

☒ All plots are contour plots with outliers or pseudocolor plots.

☒ A numerical value for number of cells or percentage (with statistics) is provided.

### Methodology

| | |
|---|---|
| Sample preparation | Described in Methods. Briefly, cells were detached using Versene, scraped and collected. The cells were washed multiple times with PBS before and after antibody treatment. They were centrifuged and the supernatant was removed for the washing process. The cells were resuspended in PBS with 5 % FBS prior to measurements |
| Instrument | BD FACSCanto II analyser |
| Software | BD FACSDiva Software, FlowJo |
| Cell population abundance | Final cell population post gating varied between 0-90% |
| Gating strategy | Live cells were selected from SSC-A vs FSC-A plots. From the live cell population, single cells were selected from FSC-H vs FSC-A plots. mScarlet/ RFP positive population was selected according to the negative non expressing control using the SSC-A vs 561 nm plots. AF647 labelled antibody binding population was selected according to the negative control expressing the construct but not uncaged via UV light using the SSC-A vs 647 nm plots. |

☒ Tick this box to confirm that a figure exemplifying the gating strategy is provided in the Supplementary Information.
